# Water fluoridation in Brazilian cities at the first decade of the 21st century

**DOI:** 10.1590/S1518-8787.2017051006372

**Published:** 2017-05-08

**Authors:** Paulo Frazão, Paulo Capel Narvai

**Affiliations:** I Departamento de Política, Gestão e Saúde. Faculdade de Saúde Pública. Universidade de São Paulo. São Paulo, SP, Brasil

**Keywords:** Fluoridation, trends, Water Supply, Public Health Policy, Oral Health

## Abstract

**OBJECTIVE:**

To assess the coverage of the fluoridation of the public water supply in Brazilian municipalities at the first decade of the 21st century, according to population size and municipal human development index (MHDI).

**METHODS:**

We have used data produced by national information agencies and the United Nations Development Programme. Population size was separated into < 10,000, 10,000–50,000, and > 50,000 inhabitants. The MHDI was classified into < 0.600, 0.600–0.699, 0.700–0.799, and > 0.799. Absolute and relative inequalities between categories were evaluated using indicators of effect and total impact.

**RESULTS:**

We have obtained information for 5,558 municipalities. The coverage rate of water fluoridation increased from 67.7% to 76.3%. Approximately 884 (15.9%) municipalities and 29,600,000 inhabitants started being benefited by the measure. We have observed a significant expansion in municipalities with < 10,000 inhabitants (increase of 21.0 percentage points) and low or very low MHDI (17.7 percentage points).

**CONCLUSIONS:**

Population coverage of the public policy has increased 8.6%, and we can also see significant reductions in absolute and relative inequality according to population size and MHDI. Regarding municipal coverage rate, there was also a reduction in inequality in all comparisons except for absolute inequality between the categories of MHDI. The public policy has operated as a factor of health protection in the context of the ongoing social protection policies in the country.

## RESUMO

**OBJETIVO:**

Avaliar a cobertura da fluoretação da água de abastecimento público em municípios brasileiros na primeira década do século XXI, segundo porte demográfico e nível de desenvolvimento humano municipal (IDH-M).

**MÉTODOS:**

Foram utilizados dados produzidos por agências nacionais de informação e pelo Programa das Nações Unidas para o Desenvolvimento. O porte demográfico foi separado em < 10 mil; 10-50 mil; > 50 mil habitantes. O IDH-M foi classificado em < 0,600; 0,600-0,699; 0,700-0,799; > 0,799. As desigualdades absoluta e relativa entre as categorias foram avaliadas por meio de indicadores de efeito e de impacto total.

**RESULTADOS:**

Foram obtidas informações para 5.558 municípios. A taxa de cobertura da fluoretação da água aumentou de 67,7% para 76,3%. Passaram a ser beneficiados pela medida 884 (15,9%) municípios, e 29,6 milhões de habitantes. Observou-se ampliação expressiva em municípios com < 10 mil habitantes (aumento de 21,0 pontos percentuais) e com IDH-M baixo ou muito baixo (17,7 pontos percentuais).

**CONCLUSÕES:**

A cobertura populacional da política pública aumentou 8,6%, sendo expressivas as reduções das desigualdades absoluta e relativa segundo o porte demográfico e o IDH-M. Quanto à taxa de cobertura municipal, houve também redução da desigualdade em todas as comparações com exceção da desigualdade absoluta entre as categorias de IDH-M. A política pública operou como fator de proteção sanitária no contexto das políticas de proteção social em curso no país.

## INTRODUCTION

Environmental conditions, notably the sanitation conditions, are a particular dimension of the development. The Human Development Index (HDI), a composite indicator used to scale the degree of human development, assigns weight to the dimensions of education, *per capita* income, and life expectancy. The impact of sanitation conditions on human development is captured only indirectly, insofar as such conditions may increase or reduce the risk of premature mortality by waterborne diseases and, consequently, the values of life expectancy. One of the goals of the HDI was to divert the focus of the development of the economy and the national income indicators to citizen-centered policies^9^. Nevertheless, references to the importance of sanitation for development and references to water are recurrent, recognized as one of the most significant determinants of health levels.

Public water supply, of special interest to public health, impacts the health of individuals and populations, both because it is essential to life, operating as a factor in health protection, and because it is a vehicle for a variety of microorganisms and, therefore, constitutes an important health risk factor^5^. An example of its action as a protection factor is the fluoridation of the public water supply, a technology widely used in successful strategies of public health. The technique of fluoridation adjusts and controls the levels of fluoride in the public water supply so that, at certain intervals, it can produce a known effect: the prevention of tooth decay. Systematic review studies have confirmed the effectiveness of preventive intervention^6,16^.

Normally, waters contain fluoride, but there is a predominance of water with poor levels, insufficient for the preventive purpose. The procedure adopted is to adjust these natural levels up to the recommended level for each location, ranging generally between 0.7 and 1.2 mg F/L. In water treatment plants, this control is done regularly and integrates the operation routine^27^. The method has been recommended by the World Health Organization (WHO)^a^ and the International Association for Dental Research (IADR)^b^, among other important organizations of international renown, for its effectiveness, safety, and low cost, which should be implemented and maintained where possible. At the beginning of the 21st century, fluoridation for caries prevention benefited approximately 400 million persons in the world^c^. As a result, the relationship between the availability of fluoride-treated water and the degree of human development in each location, region, or country are of strategic interest for public health, to the point that we can consider it a public policy by the multiplicity of interests associated with it, the complexity of the decisions involved, and the administrative and management requirements related to its implementation^19^.

Water fluoridation in treatment plants is mandatory in Brazil since 1974, according to Federal Law 6,050. Since then, the coverage of water fluoridation has been increasing^20^, and an examination of the situation of this public policy is important to evaluate the trends of expansion.

The monitoring of the coverage of health interventions in population subgroups is essential since measurements of national scope may hide important regional inequalities^3^. Studies have shown that the coverage of water fluoridation tends to be higher in larger municipalities^4,17^ and in municipalities with higher level of human development^8,19,21^. However, this notion has its origins in cross-sectional data that express the coverage of the public policy in a particular moment in the timeline. To overcome this limitation, analyses of temporal trends are essential to provide information about the dynamics of the coverage in different population subgroups.

The objective of this study is to assess the coverage of the fluoridation of the public water supply in Brazilian municipalities at the first decade of the 21st century, according to population size and human development index.

## METHODS

To calculate the absolute and relative frequencies of the municipalities that were benefited by the measure in the 2000 and 2008, we used data on the situation of the fluoridation of the public water supply, obtained from the National Survey of Basic Sanitation, carried out by the Brazilian Institute of Geography and Statistics (IBGE)^12,13^.

To analyze the changes in the implementation of the public policy, in the period between 2000 and 2008, we created four categories: (a) public policy maintained – for the municipalities that were benefited by the measure in 2000 and which, according to the information in the database, were still being benefited; (b) public policy implemented – for the municipalities that were not benefited by the measure in 2000 and which were benefited according to information recorded in 2008; (c) public policy not implemented – for those municipalities that were not benefited by the measure in 2000 and which remained without access to it in 2008; (d) public policy interrupted – for those municipalities that were benefited by the measure in 2000 and which ceased to be benefited in 2008.

Population estimates for 2000 and 2008 for each municipality provided by the IBGE and the value of the Municipal Human Development Index (MHDI) for 2000 from the United Nations Development Programme^21^ were concatenated.

For 57 municipalities, created after 2000, there was no information on population and MHDI. To not lose these municipalities, we considered the values estimated for 2002 for population and we considered the values for 2010 for MHDI, assuming as a good approximation of the population size and the level of human development for the municipality, for the purposes of this study. The information on the situation of fluoridation of these municipalities was obtained from the Brazil Water Atlas, a digital application for the visualization and analysis of indicators on the quality of the water, sanitation, and health, maintained by the *Instituto de Comunicação e Informação Científica e Tecnológica em Saúde* (Institute of Scientific and Technological Information and Communication on Health) of *Fundação Oswaldo Cruz* ^14^, in partnership with the General Coordination of Environmental Health Surveillance of the Department of Health Surveillance, Ministry of Health.

To assess the relationship between the situation of fluoridation and population size of the municipalities, population estimates for 2000 were separated into three categories: municipalities with less than 10,000 inhabitants, with 10,000 to 50,000 inhabitants, and with more than 50,000 inhabitants. This criterion has been adopted in other studies^2,24^ and took into account the current evidence indicating that the proportion of municipalities that has treated and fluoridated water is greater according to population size^4,19,21^.

To evaluate the relationship with the degree of human development, we created four categories: municipalities with MHDI below 0.600, municipalities with value between 0.600 and 0.699, municipalities with value between 0.700 and 0.799, and municipalities with MHDI above 0.799. These last three categories correspond to the categories adopted by the United Nations Development Program for average, high, and very high human development. The first category corresponds to the values that represent very low and low development^22^.

The coverages, absolute and relative, of the public policy of water fluoridation, both in terms of population and regarding the number of municipalities, were calculated considering the population estimates for each year, assuming that the records found on the situation of fluoridation are the best indication available to estimate the coverage. Absolute and relative differences between rates were measured to evaluate changes in every category of population size and human development level.

Various indicators measure health inequalities. All contain normative judgments that have important consequences for the interpretation and evaluation of health policies, and we should avoid the uncritical use of a single measure^10^. Thus, to explore the absolute and relative inequalities between the categories of analysis, we selected measures of effect and measures of total impact.

The measures of effect are useful in a stricter sense, when we want to identify trends between two population groups using as reference the best situation found. For this end, we compared the differences and the ratios of the coverage rates by adopting as the reference the category of greatest value in each year. This criterion was used to verify if the disparities between each municipality and the best situation, according to population size and level of human development, increased, decreased, or remained stable.

The measures of total impact consider all population subgroups in the measurement of inequality and are used when we want to evaluate the degree of change among all categories, covering both the best and the worst position. To that end, we used the slope index of inequality, which measures the absolute difference in the coverage rates among the categories of analysis, and the relative concentration index, which measures the percentage of change among the rates.

The slope index of inequality is calculated using a regression analysis, in which the difference among the rates corresponds to the slope of the regression line estimated by weighted least squares, representing, in this study, the change in the rate of fluoridation as the categories of analysis (population size and human development level) change from one category to another. The categories are ordered from lowest to highest (lowest to greatest population size and lowest to highest MHDI) and a score is assigned based on the average point of the cumulative distribution variation of the population corresponding to each category. For example, if the relative frequency of municipalities with MHDI < 0.600 is 15%, the variation of municipalities in this category is 0.0 to 0.15, with an average of 0.075. If the relative frequency of category of municipalities with average MHDI (0.600 to 0.699) is 30%, the variation in the cumulative distribution will be between 0.15 and 0.45, with average of 0.3, and so on. The relative concentration index belongs to the class of measures of disproportionality that express inequality as a function of the differences between the proportions of the health outcome compared with the proportions of the groups ranked with values between -1 and +1, according to the categories of analysis. Both indicators are weighted by the size of the population groups and the greater the proximity of the value to zero, the smaller the inequality^10^.

To respond to the hypothesis of the study, we sought to go beyond the knowledge produced by cross-sectional studies, analyzing if the expansion of public policy, in the period under examination, changed the disparities and in which direction, considering the analysis of not only the number of municipalities, an aspect of administrative and legal interest, but, above all, the number of inhabitants, an aspect of sanitary interest in the approach adopted.

For the statistical analysis, we used the program Stata version 12.0 (Stata Corp., College Station, United States) and HD*Calc version 1.2.4 (Health Disparities Calculator), a public access application provided by the Surveillance, Epidemiology, and End Results Program, National Cancer Institute, United States.

## RESULTS

The examination of the available data allowed us to identify 5,558 municipalities with complete information for 2000 and 2008. The data of six municipalities existing in 2008 were unavailable. Table 1 presents the distribution of inhabitants and municipalities according to population size, MHDI, and the situation of the public policy of water fluoridation between 2000 and 2008. We can observe that the public policy was expanded in the time period under study. Table 2 shows the frequency, absolute and relative, of residents and municipalities where the public policy of water fluoridation was considered as implemented in 2000 and 2008, as well as the differences between the absolute values and rates observed in each year. Removing from the municipalities that have implemented the measure (n = 989; 17.8%) those who discontinued it (n = 105; 1.9%) (Table 1), this expansion reached 884 (15.9%) municipalities which started being benefited by the measure. In the period, relative coverage for the country as a whole increased 8.6 percentage points (pp) (67.7% to 76.3% of the inhabitants). In absolute terms, this represents approximately 30 million Brazilians, according to estimates for 2008, which includes the population growth in the period (Table 2). Approximately 2,210 (39.8%) municipalities remained without access to the measure, where more than 40 million inhabitants resided in 2000 (Table 1).

**Table 1:** Absolute and relative frequency of inhabitantsa and municipalities according to population size, human development index (HDI), and the situation of the public policy of fluoridation of public water supply between 2000 and 2008.

Variable	Public policy								Total	

	Maintained		Implemented		Not implemented		Interrupted			
				
	n	%^b^	n	%^b^	n	%^b^	n	%^b^	N	%^c^
Population size										
< 10,000	5,378,315	38.6	2,840,652	20.4	5,468,785	39.2	256,321	1.8	13,944,073	8.2
	990	36.9	556	20.7	1,089	40.6	49	1.8	2,684	48.3
10,000–50,000	21,624,055	44.6	7,656,788	15.8	18,248,130	37.6	943,954	1.9	48,472,927	28.5
	1,003	42.7	390	16.6	910	38.8	45	1.9	2,348	42.2
> 50,000	85,919,442	79.8	6,352,422	5.9	14,407,122	13.4	941,029	0.9	107,620,015	63.3
	366	69.6	43	8.2	106	20.2	11	2.1	526	9.5
HDI										
< 0.600	1,135,748	10.1	2,185,648	19.5	7,752,030	69.2	135,350	1.2	11,208,776	6.6
	61	7.3	138	16.4	628	74.9	12	1.4	839	15.1
0.600–0.699	9,066,409	31.4	4,097,393	14.2	14,867,308	51.4	879,408	3.0	28,910,518	17.0
	446	26.3	281	16.5	931	54.8	40	2.4	1,698	30.6
0.700–0.799	42,874,981	64.3	8,391,689	12.6	14,421,885	21.6	1,035,735	1.6	66,724,290	39.2
	1,451	59.3	451	18.4	497	20.3	47	1.9	2,446	44.0
> 0.799	59,837,267	94.7	2,175,132	3.4	1,082,814	1.7	90,811	0.1	63,186,024	37.2
	401	69.7	119	20.7	49	8.5	6	1.0	575	10.3
Total	112,914,405	66.4	16,849,862	9.9	38,124,037	22.4	2,141,304	1.3	170,029,608	100
	2,359	42.4	989	17.8	2,105	37.9	105	1.9	5,558	100

^a^ Population estimates for 2000.

^b^ Percentages relative to row values.

^c^ Percentages relative to column values.

**Table 2:** Absolute and relative frequency of inhabitantsa and municipalities where the public policy of fluoridation of the public water supply was implemented in 2000 and 2008.

Variable	Public policy implemented				Difference between	

	2000		2008			
		
	n	%^b^	n	%^b^	Value	Rate
Population size						
< 10,000	5,627,229	40.4	10,269,250	61.4	4,642,021	21.0
	1,039	38.7	1,546	57.6	507	18.9
10,000–50,000	22,568,009	46.6	32,199,485	60.4	9,631,476	13.8
	1,048	44.6	1,393	59.3	345	14.7
> 50,000	86,860,471	80.7	102,234,834	85.5	15,374,363	4.8
	377	71.7	409	77.8	32	6.1
HDI						
< 0.600	1,271,098	11.3	3,494,837	29.0	2,223,739	17.7
	73	8.7	199	23.7	126	15.0
0.600–0.699	9,945,817	34.4	14,037,588	44.4	4,091,771	10.0
	486	28.6	727	42.8	241	14.2
0.700–0.799	43,910,716	65.8	58,108,987	77.0	14,198,271	11.2
	1,498	61.2	1,902	77.8	404	16.6
> 0.799	59,928,078	94.8	69,062,157	98.1	9,134,079	3.3
	407	70.8	520	90.4	113	19.6
Total	115,055,709	67.7	144,703,569	76.3	29,647,860	8.6
	2,464	44.3	3,348	60.2	884	15.9

HDI: Human Development Index

^a^ Population estimates for the year of reference.

^b^ Percentages relative to row values.

The magnitude of the expansion was different according to the categories of population size and human development (Table 2). As for population size, the difference in percentage points was greater in municipalities with less than 10,000 inhabitants (21.0 percentage points [pp]). Most (63.3%) of the population was distributed in 9.5% of the municipalities (those with more than 50,000 inhabitants) and 8.2% of the Brazilian population resided in municipalities with less than 10,000 inhabitants. In terms of absolute population coverage, the expansion was higher in municipalities with more than 50,000 inhabitants – approximately 15,300,000 inhabitants have been benefited by the measure, considering the population estimates for 2008.

In relation to MHDI, the class with the greatest increase in percentage points was the municipalities with very high MHDI (19.6 pp), followed by municipalities with high MHDI (16.6 pp) (Table 2). Approximately 10% of the municipalities were included in the category of very high MHDI and 15% in the categories of low or very low MHDI. In terms of population, the category of municipalities with very low and low MHDI, a class which comprised 6.6% of the inhabitants, showed the highest increase in percentage points (17.7 pp) of the population who was benefited by the measure. The increase in absolute population coverage was higher in the category of municipalities with high MHDI, in which more than 14.1 million inhabitants were benefited by the measure, considering the population estimates for 2008 (Table 2).

As for municipalities, the percentage of municipalities in which the public policy was implemented increased from 44.3% to 60.2%, corresponding to an increase of 15.9 pp and, in absolute terms, a difference of 884 municipalities, of which 45.7% (404) were municipalities with high MHDI and 23.7% (241) were municipalities with average MHDI (Table 2).

Table 3 presents the values corresponding to the differences and the ratios of population coverage rates between the municipalities of different population sizes and human development levels, taking as reference municipalities with more than 50,000 inhabitants and very high MHDI. The comparison of the values obtained in 2000 and 2008 indicates a significant reduction of inequalities, both absolute and relative, in the comparison of each municipality with the reference category, in the total change, population coverage, and the coverage of the number of municipalities according to the categories of population size and MHDI. In relation to population size, the percentage of reduction of total absolute inequality was 31.6% and the percentage of reduction of total relative inequality was 39.0%, being the greatest reductions found in the comparison between the categories of lower and greater population size. In relation to MHDI, the percentage of absolute and relative reduction were, respectively, 15.9% and 28.6%, being the greatest reductions found in the comparison between the municipalities of average MHDI and the reference category (in absolute terms), and in the comparison between the municipalities of very low or low MHDI and the reference category (in relative terms).

**Table 3:** Absolute and relative inequality values obtained from the population coverage rates of fluoridation of the public water supply for 2000 and 2008.

Variable	Population coverage					

	Absolute difference between rates			Relative difference between rates		
	
	2000	2008	% change	2000	2008	% change
Population size						
< 10,000	40.3	24.1	-40.2	2.0	1.4	-30.0
10,000–50,000	34.1	25.1	-26.4	1.7	1.4	-17.6
> 50,000	Reference			Reference		
	Slope index of inequality			Relative concentration index		
	69.7	47.6	-31.6	0.12	0.08	-39.0
HDI						
< 0.600	83.5	69.1	-17.3	8.4	3.4	-59.5
0.600–0.699	60.4	53.7	-11.1	2.8	2.2	-21.4
0.700–0.799	29.0	21.1	-27.2	1.4	1.3	-7.1
> 0.799	Reference			Reference		
	Slope index of inequality			Relative concentration index		
	94.1	79.2	-15.9	0.21	0.15	-28.6

HDI: Human Development Index

In Table 4 we show the values concerning the municipal coverage rates. In relation to the categories of population size, the comparison between 2000 and 2008 indicated a significant reduction of the absolute (47.1%) and relative (66.6%) inequality. In relation to the categories of human development, we observed an increase in absolute inequality (6.6%) and a reduction in relative inequality (23.1%), both in the total change and in the comparison of each municipality with the reference category.

**Table 4:** Absolute and relative inequality values obtained from the municipal coverage rates of fluoridation of the public water supply for 2000 and 2008.

Variable	Municipal coverage					

	Absolute difference between rates			Relative difference between rates		
	
	2000	2008	% change	2000	2008	% change
Population size						
< 10,000	33.0	20.2	-38.8	1.9	1.3	-31.6
10,000–50,000	27.1	18.4	-32.1	1.6	1.3	-18.8
> 50,000	Reference			Reference		
	Slope index of inequality			Relative concentration index		
	28.1	14.9	-47.1	0.09	0.03	-66.6
HDI						
< 0.600	62.1	66.7	7.4	8.1	3.8	-53.1
0.600–0.699	42.2	47.6	12.8	2.5	2.1	-16.0
0.700–0.799	9.6	12.6	31.3	1.2	1.2	0.0
> 0.799	Reference			Reference		
	Slope index of inequality			Relative concentration index		
	78.5	83.7	6.6	0.26	0.20	-23.1

HDI: Human Development Index

## DISCUSSION

Considering that the HDI does not directly address the dimension of environmental conditions and, in them, the provision of treated water, studies that, as this one, seek these two dimensions are relevant, as they try to connect them to produce relevant knowledge for the evaluation of the implementation of public policies. In addition, public policies, in the case of health, apart from their specific purposes, must operate to reduce inequalities. Although this effect is not obtained in all situations, being frequent the cases in which the implementation of certain actions results in increased inequalities and not vice versa, we need to analyze concrete situations of implementation of these policies, so that, with such knowledge, the implementation can be redirected or even ceased.

The results showed that, between 2000 and 2008, 8.6% of the Brazilian population started receiving the benefit, increasing the coverage rate from 67.7% to 76.3%, one of the highest coverage among the ten most populous countries of the planet. In addition, we observed an expansion of water fluoridation in all population subgroups, both from the point of view of population coverage and the number of municipalities benefited.

In 2000, the proportion of municipalities benefit by the public policy of water fluoridation was approximately two times lower in municipalities with up to 10,000 inhabitants when this set of municipalities was compared with the set represented by municipalities with 50,000 inhabitants or more, corroborating the value obtained from a national research from a sample of 250 municipalities^17^. In 2008, the relative inequality decreased to 1.4. The significant expansion in the relative coverage of the population in municipalities with less than 10,000 inhabitants (increase of 21.0 pp) and with low or very low MHDI (17.7 pp) and the reduction of total, absolute, and relative inequality, among the subgroups in 2000 and 2008, suggest a different scenario from the knowledge produced by previous studies^4,8,17,19,21^, indicating that Brazil seems to be overcoming a characteristic of the implementation of this measure that marked it in the second half of the 20th century.

In a classic study, Hart^11^ has shown that, if implemented under the laws of the market, health actions often benefit more the population better situated on the socioeconomic scale. Contextualizing this assertion for the maternal and child health, researchers have shown that good public health programs end up being more used at first by those who least need them^26^. This leads us to think that certain interventions, even under the management of the government and apparently immune to the laws of the market, when implemented in the context of high socioeconomic inequality, would tend to benefit the richest first, and only some time later, the poorest.

In the case of fluoridation of the public water supply, it is important to consider that a necessary condition for its implementation is the existence of a system or network of water treatment and distribution. In a municipality where the network reaches the richest and the poorest areas, its application represents a factor of immediate protection to all households connected to the network, regardless of the socioeconomic condition of each family. In relation to the Brazilian case – a context of high socioeconomic inequality –, it is pertinent to point out that the differences in the distribution of the benefit observed among the municipalities in the second half of the 20th century^8,17,21^ took place largely from the sanitation model adopted by the country, in which the distribution and access to services were not an example of equity^18^. A strong business concept predominated in which investments were directed to yield high profitability and rapid return of the capital invested^15^.

Despite this important expansion of population coverage precisely in the segments most in need of access to this benefit, it is worth mentioning that, from the 884 municipalities that began having this public policy in 2008, 45.7% (404) were municipalities with high MHDI and 23.7% (241) with average MHDI, a change that increased by 6.6% the total absolute inequality between the municipal coverage rates, which means that the public policy remains reproducing the “law of Hart” in favor of the municipalities with better human development level.

In addition, 105 municipalities ceased to be benefited by the measure, of which 94 were municipalities with less than 50,000 inhabitants and 87 had average or high human development, making us wonder about what would be the reasons that would have led to this interruption. Considering that the interruption of this public policy can be technically unjustified, illegal, and ethically unfair^20^, we need to investigate which measures or procedures are being adopted by the authorities and the communities to question those responsible and act to reverse the interruption.

Among the limitations of this study, it is worth pondering the values of health intervention coverage. We assume some degree of imprecision, as the data available are the result of processes not fully validated using appropriate techniques^7^. We may have overestimated the values in relation to population coverage, as the number of inhabitants residing in rural areas is reached only indirectly by the public policy. Additionally, the overestimation can come from municipal coverages, as the situation of the fluoridation was obtained by the IBGE using a questionnaire with the entities that provide sanitation services without mention of any other source of information^12,13^. Considering that the outcomes resulting from this preventive measure can be measured only a few years after implementation, experts have recommended that the control of the fluoridation should be done by agencies not directly responsible for the water treatment (principle of external control), using the direct assessment of water samples collected from the distribution network^20,25^. In this way, the quality of the process, the validity of the information, and the reliability is ensured to achieve the goals of oral health. In addition, in many Brazilian municipalities, only the populations of urban centers have access to treated water.

We could argue that a similar study, using distinctive criterion of classification of municipalities according to population size, could produce results other than those found in this research. In fact, it is always difficult to establish criteria which allow a balanced composition of the categories according to population size. Most Brazilian municipalities have a very small fraction of the total population. On the one hand, approximately half of the Brazilian municipalities have little more than 10,000 inhabitants, and the other hand, 1/3 of the total population lives in 56 municipalities. To explore this aspect, we calculated the values of absolute and relative inequality related to total impact and effect indicators, adopting two other possibilities of classification of the municipalities. At first, the municipalities were divided into tertiles according to population size, that is, a tertile of the less populous, an intermediate tertile, and a tertile of the most populous municipalities. In the second classification, the municipalities were grouped according to the tertile of accumulated frequency of the population, i.e. each of the tertiles concentrated 1/3 of the population. Small variations were noted, but there was no change in the direction of the inferences in any of the situations mentioned, confirming the results shown in this study.

As a result, we can conclude that, in the period under examination, the coverage rate of water fluoridation increased 8.6%, with significant expansion in municipalities with less than 10,000 inhabitants (21.0 pp) and with low and very low MHDI (17.7 pp). Between 2000 and 2008, we could observe a significant reduction of the total relative inequality, both in terms of population coverage and the coverage of the number of municipalities according to the categories of population size and MHDI. Total absolute inequality also decreased, except for municipal coverage rates according to the categories of human development. According to official data, in the period from 2005 to 2008, 711 new systems of fluoridation were installed in 503 municipalities, in 11 States, benefiting 7,600,000 Brazilians^1,23^. The results observed suggest that the efforts made by both the federal level and the State level were not enough to accelerate the expansion of the measure in municipalities with very low or low MHDI and implementation strategies need to be readjusted.

Nevertheless, the public policy of fluoridation showed a performance compatible with the requirements to produce health benefits and, at the same time, contribute to the reduction of inequalities. With that, the public water supply, of special interest to public health, operated in the Brazilian scenario, as described in this article, as a health protection factor, in the context of ongoing social protection policies in the country.

## Footnotes

a World Health Organization. Resolution WHA60.1. In: the Sixtieth World Health Assembly; 2007 May 26; Geneva, Swiss. Geneva: WHO; 2007 [cited 2015 Oct 27]. Available from: http://apps.who.int/gb/ebwha/pdf_files/WHASSA_WHA60-Rec1/E/reso-60-en.pdf

b The Liverpool Declaration: Promoting Oral Health in the 21st Century. Geneva: WHO; 2007 [cited 2015 Oct 27]. Available from: http://www.who.int/oral_health/events/orh_liverpool_declaration_05.pdf

c The British Fluoridation Society. Extent of water fluoridation. Oldham: BFS; s.d. [cited 2015 Oct 27]. Available from: https://www.bfsweb.org/extent-of-water-fluoridation
